# The characteristics and antigenic properties of recently emerged subclade 3C.3a and 3C.2a human influenza A(H3N2) viruses passaged in MDCK cells

**DOI:** 10.1111/irv.12447

**Published:** 2017-02-28

**Authors:** Yipu Lin, Stephen A. Wharton, Lynne Whittaker, Mian Dai, Burcu Ermetal, Janice Lo, Andrea Pontoriero, Elsa Baumeister, Rodney S. Daniels, John W. McCauley

**Affiliations:** ^1^ The Francis Crick InstituteLondonUK; ^2^ Centre for Health ProtectionDepartment of HealthHong Kong Special Administrative RegionChina; ^3^ Instituto Nacional de Enfermedades Infecciosas‐ANLIS “Dr.Carlos G.Malbran”Buenos AiresArgentina

**Keywords:** antigenicity, influenza, MDCK cells, MDCK‐SIAT1 cells, receptor binding

## Abstract

**Background:**

Two new subclades of influenza A(H3N2) viruses became prominent during the 2014‐2015 Northern Hemisphere influenza season. The HA glycoproteins of these viruses showed sequence changes previously associated with alterations in receptor‐binding properties. To address how these changes influence virus propagation, viruses were isolated and propagated in conventional MDCK cells and MDCK‐SIAT1 cells, cells with enhanced expression of the human receptor for the virus, and analysed at each passage.

**Methods:**

Gene sequence analysis was undertaken as virus was passaged in conventional MDCK cells and MDCK‐SIAT1 cells. Alterations in receptor recognition associated with passage of virus were examined by haemagglutination assays using red blood cells from guinea pigs, turkeys and humans. Microneutralisation assays were performed to determine how passage‐acquired amino acid substitutions and polymorphisms affected virus antigenicity.

**Results:**

Viruses were able to infect MDCK‐SIAT1 cells more efficiently than conventional MDCK cells. Viruses of both the 3C.2a and 3C.3a subclades showed greater sequence change on passage in conventional MDCK cells than in MDCK‐SIAT1 cells, with amino acid substitutions being seen in both HA and NA glycoproteins. However, virus passage in MDCK‐SIAT1 cells at low inoculum dilutions showed reducing infectivity on continued passage.

**Conclusions:**

Current H3N2 viruses should be cultured in the MDCK‐SIAT1 cell line to maintain faithful replication of the virus, and at an appropriate multiplicity of infection to retain infectivity.

## 1 Introduction

Influenza A(H3N2) viruses have, since their introduction into humans in 1968 as “Hong Kong flu,” undergone extensive evolution both genetically and antigenically causing numerous seasonal influenza epidemics and warranting the WHO‐recommended H3N2 component of vaccines to be changed 28 times. Over their nearly 50‐year history in humans, H3N2 viruses have also altered their receptor‐binding properties^1^, ^2^, ^3^, ^4^, ^5^ with a progressive reduction in recognition of oligosaccharide analogues of cell surface receptors^1^. Amino acid substitutions at two key positions in the haemagglutinin (HA), residues 222 and 225 of the large polypeptide chain of the haemagglutinin (HA1), have occurred in H3N2 viruses in circulation since 2002 and shown to be associated with a progressive decrease in binding to analogues of the receptors recognised by human influenza viruses^1^. In comparison with the structure of the HA from a 1968 virus, carrying 222W and 225G in HA1, the 220 loops of viruses from both 2004, with HA1 222R and 225D, and 2005, with 222R and 225N, have become displaced by 1.5Å. Changes in the receptor‐binding affinity of these viruses can be explained by the observations that the 220 loop of a 2004 HA moves to adopt the conformation of that of the 1968 prototype on binding the receptor analogue with 225D forming a hydrogen bond with Gal‐2 of the receptor analogue, while the 220 loop of the HA of a virus that carries 225N does not undergo a conformational change on receptor binding and is unable to form this hydrogen bond^1^.

Associated with the progressive changes in receptor binding are changes in the abilities of circulating viruses to infect cells in culture. Viruses isolated after 2001 display decreased ratios of infection of conventional Madin Darby canine kidney (MDCK) cells in comparison with MDCK‐SIAT1 cells, cells modified to express a higher density of α2,6 human receptors^4^, ^6^, ^7^. Strikingly, the viruses with the lowest avidity for the human receptor showed the greatest reductions in their ability to infect MDCK cells relative to MDCK‐SIAT1 cells^1^. Moreover, isolation and passage in MDCK cells of H3N2 viruses collected since 2005 can lead to the selection of a virus population in which amino acid polymorphism or substitutions at residues 148 or 151 in the virus neuraminidase (NA) complements the HA in receptor binding, a property inhibited by the addition of oseltamivir^6^.

Since their first detection in late March 2014, two new H3N2 virus variants established themselves rapidly and became the predominant virus subtype circulating in North America and Europe during the 2014‐2015 Northern Hemisphere winter season^8^. The new variants fell in phylogenetic clades 3C.2 and 3C.3 and formed subclades 3C.2a and 3C.3a. Amino acid substitutions that define these subclades compared with a previously used vaccine virus, A/Texas/50/2012, are as follows: (3C.2a) L3I, N144S (resulting in the loss of a potential glycosylation site), N145S, F159Y, K160T (resulting in the gain of a potential glycosylation site), N225D and Q311H in HA1; (3C.3a) T128A (resulting in the loss of a potential glycosylation site), A138S, R142G, N145S, F159S and N225D in HA1. Both new variants have substitutions in antigenic sites and glycosylation sites of their HAs but, notably, viruses in both subclades encoded aspartic acid at residue 225 of HA1 (225D).

In the light of the reversion at residue 225 of HA1, to that of viruses in circulation in humans from 2002 to 2004, it seemed possible that the recognition of receptor by the 3C.2a and 3C.3a viruses had altered. Notably, changes in receptor recognition can affect the efficiency of virus propagation in cell culture^1^, ^4^. Recently, it has been reported that subclade 3C.2a viruses can acquire polymorphism on culture^9^. To extend these recent observations, we have examined the propagation of H3N2 viruses from subclades 3C.2a and 3C.3a in conventional MDCK cells and in MDCK‐SIAT1 cells, and analysed sequence changes acquired on culture in the HA and NA glycoproteins. We further assessed whether amino acid substitutions acquired during propagation altered the antigenic and receptor‐binding properties of the new subclades of virus.

## 2 Materials and Methods

### 2.1 Cells

MDCK‐SIAT1 cells, stably expressing human CMP‐N‐acetylneuraminate:β‐galactoside α‐2,6‐sialyltransferase^7^ and the conventional MDCK cell line from which they were derived (MDCK‐parent), were kindly provided by Dr. M. Matrosovich, Philipps‐Universität, Marburg, Germany. Cells were propagated in Dulbecco's modified Eagle's medium (DMEM (Sigma D6429)), supplemented with 10% (v/v) heat‐inactivated foetal calf serum (FCS), penicillin (100 U/mL) and streptomycin (100 μg/mL) at 37°C with 5% (v/v) CO_2_. G418 sulphate (Geneticin; GIBCO) (1 mg/mL) was added to the culture medium for propagating MDCK‐SIAT1 cells.

### 2.2 Clinical specimens

The clinical specimens used in this study were collected during the 2013‐2014 influenza season and provided by the WHO National Influenza Centres in Hong Kong SAR, China, and Buenos Aires, Argentina.

### 2.3 Virus isolation and propagation

Hundred microlitres per well of clinical specimens were inoculated in parallel on confluent cell monolayers of MDCK‐parent or MDCK‐SIAT1 cells in 24‐well tissue culture plates and incubated at room temperature for 1 hour, and then, 1 mL of DMEM supplemented with 2 μg/mL TPCK‐treated trypsin (Sigma Cat no T1426) was added. Cultures were incubated at 34°C with 5% (v/v) CO_2_ for 72 hour. Further passages were performed using 200 μL inocula of cell culture supernatant per well, both undiluted and at higher dilution, from the previous passage.

### 2.4 Haemagglutination assays (HAA)

HAA were performed according to standard methods^10^ using suspensions of guinea pig, human (both 1.0% v/v) or turkey (0.75% v/v) red blood cells (RBC), and HAA titres were determined in the absence or presence of 20 nM oseltamivir carboxylate in the diluent^6^.

### 2.5 Neuraminidase activity quantification

Sialidase activity of each virus was measured using 2′‐(4‐methylumbelliferyl)‐α‐D‐N‐acetyl neuraminic acid (MUNANA; Sigma)^11^. The dilution factor of the virus required to give 50% conversion of the total MUNANA substrate, at an initial concentration of 60 μM, over 60 minutes at 37°C was taken as a relative measure of the sialidase content of the sample.

### 2.6 Nucleotide sequence analysis

Following RT‐PCR, nucleotide sequences of HA and NA genes were determined by Sanger dideoxynucleotide sequencing using ABI prism BigDye terminator cycle sequencing kits (Cat. No. 4336774) and an ABI 3730XL DNA analyser. Primer sequences are available on request. Next‐generation sequencing (NGS) was performed using a MBT‐universal 3 primer approach^12^, with Illumina Nextera XT (Cat nos. FC‐131‐1096, FC‐131‐1002) sample preparation and indexing, on an Illumina MiSeq sequencer.

### 2.7 Plaque and Microneutralisation (MN) assays

These were performed as described previously^13^, ^14^.

## 3 Results

### 3.1 Detection of amino acid substitutions or polymorphisms in HA and NA following passage in MDCK cells

Clinical specimens were inoculated onto conventional MDCK cells or MDCK‐SIAT1 cells, and viruses were passaged twice further in each cell line. The HA and NA gene sequences of viruses in the clinical specimens were determined by both Sanger and deep sequencing methods, and viruses from each tissue culture passage were sequenced using Sanger sequencing. The results of Sanger sequencing are summarised in Table 1. For subclade 3C.3a viruses, only minor polymorphism at HA1 positions 158‐160 was detected in the three clinical specimens analysed by NGS and any polymorphism seen was only observed at residue 159. Sanger sequencing on five passaged viruses of the 3C.3a clade from either the conventional MDCK cell line or the MDCK‐SIAT1 cell line showed no polymorphism in this region of the HA (Table 1).

**Table 1:** Amino acid substitutions in HA and NA of subclade 3C.3a and 3C.2a viruses after propagation in conventional MDCK cells or MDCK‐SIAT1 cells

Virus name	Subclade	Passage^a^	Clinical specimen		Passage 1		Passage 2		Passage 3	
			HA 158‐160	NA 148/151	HA 158‐160	NA 148/151	HA 158‐160	NA 148/151	HA 158‐160	NA 148/151
A/Hong Kong/6033/2014	3C.3a	cs	?^b^	?						
		MDCK‐p			NSK	151D	NSK	151D	NSK	(151D50:N50)^c^
		SIAT1			NSK	151D	NSK	151D	NSK	151D
A/Hong Kong/6086/2014		cs	NSK	151D						
		MDCK‐p			NSK	151D	NSK	151D	NSK	(151D80:N20)
		SIAT1			NSK	151D	NSK	151D	NSK	151D
A/Hong Kong/6315/2014		cs	?	?						
		MDCK‐p			NSK	151D	NSK	151D	NSK	(151D60:N40)
		SIAT1			NSK	151D	NSK	151D	NSK	151D
A/Hong Kong/6421/2014		cs	NSK	151D						
		MDCK‐p			NSK	151D	NSK	151D	NSK	151D
		SIAT1			NSK	151D	NSK	151D	NSK	151D
A/Hong Kong/6872/2014		cs	NSK	151D						
		MDCK‐p			NSK	151D	NSK	(151D50:N50)	NSK	(151D60:N40)
		SIAT1			NSK	151D	NSK	151D	NSK	151D
A/Hong Kong/6013/2014	3C.2a	cs	NYT	151D						
		MDCK‐p			NYT	151D	NYT	(151D80:N20)^c^	NYT	(151D50:N50)
		SIAT1			NYT	151D	NYT	151D	NYT	151D
A/Hong Kong/7127/2014		cs	NYT	151D						
		MDCK‐p			NYT	151D	NY(T20:A80)	(151D80:G20)	NY(T20:A80)	(151D50:N50)
		SIAT1			NYT	151D	NYT	151D	NYT	151D
A/Hong Kong/7278/2014		cs	NYT	151D						
		MDCK‐p			NYT	151D	NYT	151D	**NYT**	(151D75:N25)
		SIAT1			NYT	151D	NYT	151D	NYT	151D
A/Hong Kong/7295/2014		cs	NYT	151D						
		MDCK‐p			NYT	151D	NYT	(148T50:K50) 151D	NYT	(148T60:K40 151D50:N50)
		SIAT1			NYT	151D	NYT	151D	NYT	148T 151D
A/Hong Kong/7347/2014		cs	?^b^	?						
		MDCK‐p			?	151D	NFT	151D	NF(T30:A70)	151D
		SIAT1			NFT	151D	NFT	151D	NFT	151D
A/Hong Kong/7364/2014		cs	NYT	151D						
		MDCK‐p			NYT	151D	(K60:N40)YT	(148T70:I30) 151D	(K60:N40)YT	(148T70:K30 151D50:N50)
		SIAT1			NYT	151D	NYT	151D	NYT	151D
A/Hong Kong/5576/2014		cs	NYT	151D						
		MDCK‐p			NYT	151D	NYT	151D	NY(K70:T30)	(151D85:G15)
		SIAT1			NYT	151D	NYT	151D	NYT	151D
A/Buenos Aires/2274920/2014		cs	?	?						
		MDCK‐p			NYT	151D	NYT	(148K70:T30) 151D	NYT	(148K70:T30) 151D
		SIAT1			NYT	151D	NYT	151D	NYT	151D
A/Buenos Aires/2275130/2014		cs	?	?						
		MDCK‐p			NYT	151D	NYT	151D	NYT	(151D60:V40)
		SIAT1			NYT	151D	NYT	151D	NYT	151D

a Indicates cell line used for propagation: MDCK‐p=conventional MDCK‐parent cells, SIAT1=MDCK‐SIAT1 cells; cs=clinical specimen.

b ?=Sequence not available.

c Relative proportions of amino acids at polymorphic positions.

In contrast, for six of seven clinical specimens in subclade 3C.2a, NGS data showed increased variation in the HA gene encoding amino acids at positions 158‐160 of HA1, notably so at residue 160 (Table S1). On passage of virus isolates on conventional MDCK cells using undiluted supernatants from the previous passage, results from Sanger sequencing showed four of nine subclade 3C.2a viruses to have acquired considerable polymorphism at residues 158 or 160 by passage three. Such polymorphism was not seen when MDCK‐SIAT1 cells were used for virus isolation and propagation (Table 1).

Both subclade 3C.3a and 3C.2a viruses showed polymorphism at NA positions 148 or 151 after three passages in conventional MDCK cells (four of five subclade 3C.3a and eight of nine subclade 3C.2a viruses), but no similar polymorphism in the NA was detected in viruses propagated in MDCK‐SIAT1 cells (Table 1).

### 3.2 Binding of viruses in subclades 3C.2a and 3C.3a to erythrocytes from different species

Receptor‐binding properties of viruses in the two new subclades were assessed by their abilities to agglutinate erythrocytes from turkeys, guinea pigs and humans. The results are shown in Table 2. Subclade 3C.3a viruses propagated in MDCK‐SIAT1 cells were able to bind to guinea pig RBC (GPRBC) regardless of whether oseltamivir was present or not. In contrast, no haemagglutination with GPRBC was observed for any of the 3C.2a viruses propagated in MDCK‐SIAT1 cells. Viruses in both subclades 3C.3a and 3C.2a were unable to agglutinate turkey RBC (TRBC) or human RBC (HRBC) when 20 nM oseltamivir carboxylate was included in the assay, with the exception of A/Hong Kong/6315/2014 (3C.3a) which had a titre of 2 after the first passage. In summary, for viruses propagated in MDCK‐SIAT1 cells, despite virus plaque‐forming units (PFU) at titres of over 10^5^ PFU/mL and high NA activities (over 25,000 relative fluorescence units (RFU)), only viruses in subclade 3C.3a were able to bind GPRBCs.

**Table 2:** Haemagglutination of subclade 3C.3a and 3C.2a viruses with RBC from three species

Virus name	Subclade	Passage^a^	Passage 1						Passage 2								Passage 3							
			RBC^b^				PFU/mL	NA^c^	RBC^b^						PFU/mL	NA^c^	RBC^b^						PFU/mL	NA^c^
			T−	T+	G−	G+			T−	T+	G−	G+	H−	H+			T−	T+	G−	G+	H−	H+		
A/Hong Kong/6033/2014	3C.3a	MDCK‐p	<^d^	<	8‐16	4‐8	10^8^	pos	<	<	16	8	2	<	6×10^7^	pos	32	<	64	16	16	<	1×10^7^	pos
		SIAT1	<	<	8	8	6×10^7^	pos	<	<	8	8	<	<	5×10^7^	pos	<	<	16	16	<	<	1×10^7^	pos
A/Hong Kong/6086/2014		MDCK‐p	<	<	8	8	3×10^7^	pos	<	<	16	8	2	<	3×10^7^	pos	16	<	32	16	32	<	4×10^5^	pos
		SIAT1	<	<	32	16	3×10^7^	pos	<	<	8	4	<	<	8×10^5^	pos	<	<	16	8	<	<	2×10^3^ sin^e^	pos
A/Hong Kong/6315/2014		MDCK‐p	<	<	8‐16	8‐16	1.6×10^6^	pos	<	<	16	8	<	<	2×10^7^	pos	16	<	32	16	16	<	3×10^5^	pos
		SIAT1	2	2	32	32	4×10^6^	pos	<	<	16	16	<	<	4×10^5^	pos	<	<	16	16	<	<	2×10^3^ sin	pos
A/Hong Kong/6421/2014		MDCK‐p	<	<	<	<	8×10^3^	neg	<	<	8	4	<	<	5×10^7^	pos	<	<	32	32	2	2	2.2×10^7^	pos
		SIAT1	<	<	<	<	5×10^6^	low	<	<	16	8	<	<	4×10^6^	pos	<	<	16	16	<	<	6×10^4^ sin	pos
A/Hong Kong/6872/2014		MDCK‐p	2	<	4‐8	2‐4	2×10^7^	pos	64	<	64	4	64	<	3×10^7^	pos	32	<	64	8	64	<	1.6×10^5^	pos
		SIAT1	<	<	4‐8	4‐8	3×10^7^	pos	<	<	4	2	<	<	1×10^7^	pos	<	<	8	8	<	<	6×10^4^ sin	pos
A/Hong Kong/6013/2014	3C.2a	MDCK‐p	<	<	<	<	1×10^6^	low	<	<	<	<	<	<	3×10^7^	pos	32	<	64	2	64	<	2×10^7^	pos
		SIAT1	<	<	<	<	1×10^7^	pos	<	<	<	<	<	<	1×10^6^	pos	<	<	2	2	<	<	2×10^3^ sin	pos
A/Hong Kong/7127/2014		MDCK‐p	<	<	<	<	1×10^7^	pos	8	<	16	4	16	4	4×10^7^	pos	32	<	16	4	16	2	2×10^6^	pos
		SIAT1	<	<	<	<	1×10^7^	pos	<	<	<	<	<	<	5×10^6^	pos	<	<	<	<	<	<	1.4×10^5^ sin	pos
A/Hong Kong/7278/2014		MDCK‐p	<	<	<	<	8×10^6^	low	<	<	<	<	<	<	2×10^7^	pos	16	<	16	4	16	4	1.2×10^7^	pos
		SIAT1	<	<	<	<	1×10^7^	pos	<	<	<	<	<	<	1×10^6^	pos	<	<	<	<	<	<	6×10^4^ sin	pos
A/Hong Kong/7295/2014		MDCK‐p	<	<	<	<	8×10^5^	low	<	<	8	<	<	<	3×10^7^	pos	32	<	32	<	32	<	1×10^7^	pos
		SIAT1	<	<	<	<	3×10^7^	pos	<	<	<	<	<	<	1×10^7^	pos	<	<	<	<	<	<	1×10^6^	pos
A/Hong Kong/7347/2014		MDCK‐p	<	<	<	<	1×10^5^	low	<	<	<	<	<	<	1×10^7^	pos	<	<	4	4	<	<	2×10^7^	pos
		SIAT1	<	<	<	<	4×10^7^	pos	<	<	<	<	<	<	3×10^7^	pos	<	<	<	<	<	<	1.4×10^7^	pos
A/Hong Kong/7364/2014		MDCK‐p	<	<	<	<	2×10^6^	pos	32	8	32	8	32	8	3.2×10^7^	pos	64	4	64	8	64	8	1.6×10^6^	pos
		SIAT1	<	<	<	<	2.4×10^7^	pos	<	<	<	<	<	<	8×10^5^	pos	<	<	<	<	<	<	2×10^2^ sin	pos
A/Hong Kong/5576/2014		MDCK‐p	<	<	<	<	5×10^5^	low	<	<	<	<	<	<	4×10^6^	pos	64	16	64	16	64	32	2×10^7^	pos
		SIAT1	<	<	<	<	8.8×10^7^	pos	<	<	<	<	<	<	8×10^6^	pos	<	<	<	<	<	<	1.6×10^5^	pos
A/Buenos Aires/2274920/2014		MDCK‐p	<	<	<	<	4×10^5^	neg	32	<	32	<	16	<	5×10^7^	pos	64	<	32	<	32	<	1.6×10^7^	pos
		SIAT1	<	<	<	<	5×10^7^	pos	<	<	<	<	<	<	1.6×10^7^	pos	<	<	<	<	<	<	1.6×10^6^	pos
A/Buenos Aires/2275130/2014		MDCK‐p	<	<	<	<	5.2×10^5^	low	<	<	<	<	<	<	4×10^6^	pos	64	<	64	2	64	2	1×10^7^	pos
		SIAT1	<	<	<	<	6.8×10^7^	pos	<	<	<	<	<	<	1×10^7^	pos	<	<	<	<	<	<	2.4×10^5^	pos

a Indicates cell line used for propagation: MDCK‐p=conventional MDCK‐parent cells, SIAT1=MDCK‐SIAT1 cells.

b T, G and H indicate different RBC used in the haemagglutination assay: T=Turkey, G=Guinea pig and H=Human. 20 nM oseltamivir carboxylate included (+) or absent (−) in the assays.

c Neuraminidase activity determined by relative fluorescence units: pos=>25 000, low=4000‐25 000, neg=<4000.

d <=<2.

e sin=single cell infection.

Viruses propagated in conventional MDCK cells showed different binding profiles. Following three passages, four of five subclade 3C.3a viruses and eight of nine subclade 3C.2a viruses bound to all three species of RBC tested; however, HA titres were significantly reduced when 20 nM oseltamivir was included in the assay. These results are consistent with the binding of virus to RBC being mediated by NA, as reported previously for cell culture‐propagated H3N2 viruses^6^, and are indicative of the continued culture‐driven selection of viruses in both subclades in conventional MDCK cells.

### 3.3 Infection of conventional MDCK and MDCK‐SIAT1 cells by recent H3N2 viruses

In the light of previous observations that recent H3N2 viruses are more readily propagated in the MDCK‐SIAT1 cell line than in conventional MDCK cells^1^, ^4^ the ability of viruses in the newly emerged subgroups to infect MDCK‐SIAT1 and conventional MDCK cells was examined.

**Figure 1:**
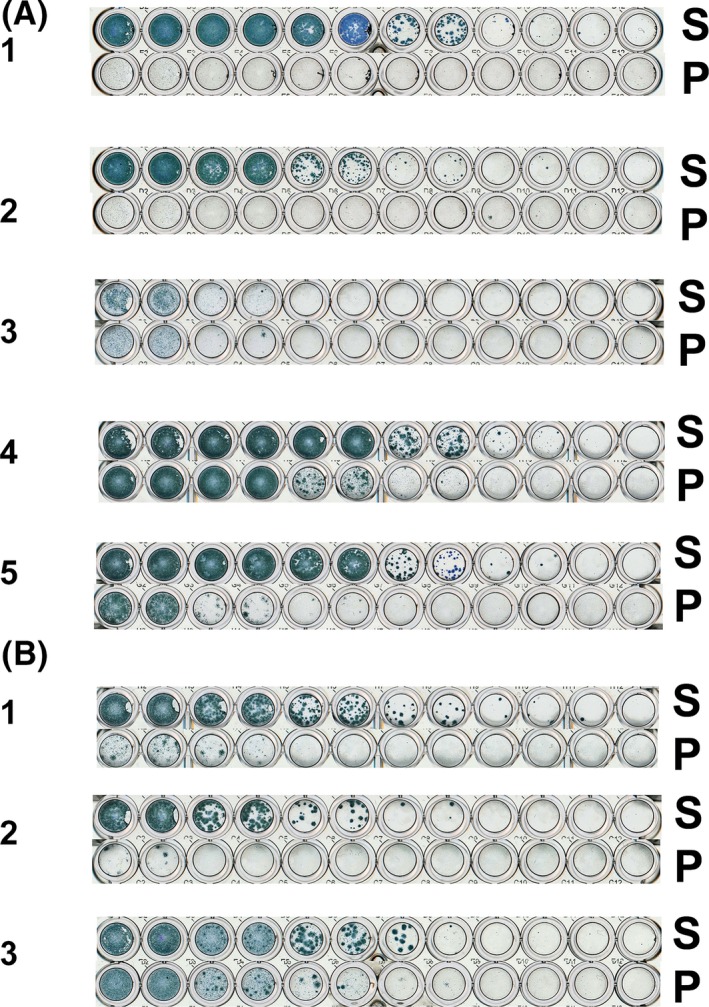
Comparison of plaque formation by 3C.2a and 3C.3a viruses on MDCK‐SIAT1 and MDCK‐parent cells. Plaque titrations were performed in parallel using MDCK‐SIAT1 (S) and MDCK‐parent (P) cells in duplicate with 10‐fold dilutions. Panel A: 3C.2a viruses: 1, A/Asturias/1951/2014 and 2, A/Buenos Aires/6367/2014 both retained the glycosylation site at HA1 158‐160, viruses with no polymorphism in NA 148/151; 3, A/Israel/O‐7414/2014, with no glycosylation site due to a T160K substitution in HA1 and no polymorphism at NA 148/151; 4, A/Hong Kong/7347/2014 with a partially lost glycosylation site due to polymorphism at HA1 160 (K/T) and no polymorphism at NA 148/151; 5, A/Hong Kong/6013/2014 retained glycosylation site at HA 158‐160 but with polymorphism at NA 148/151. Panel B: 3C.3a viruses: 1, A/Switzerland/9715293/2013 and 2, A/Norway/466/2014 have no polymorphism in NA 148/151; 3, A/Hong Kong/6315/2014 with polymorphism in NA 151. Viruses with polymorphisms were from passage 3 using conventional MDCK cells, and the extents of polymorphisms are as shown in Table 1

The relative abilities of five 3C.2a and three 3C.3a viruses to form virus plaques/foci of infection were examined in conventional MDCK cells and MDCK‐SIAT1 cells (Figure 1). The 3C.2a viruses examined were chosen to exemplify viruses that had retained the HA1 158‐160 glycosylation motif, A/Asturias/1951/2014 and A/Buenos Aires/6367/2014; that had no HA1 158‐160 glycosylation motif, A/Israel/O‐7414/2014; or had, on culture, acquired polymorphism in the HA1 158‐160 glycosylation motif, A/Hong Kong/7347/2014; and a virus that retained the HA1 158‐160 glycosylation motif but had acquired polymorphism in the NA at residue 151 leading to NA complementation of the HA in receptor binding, A/Hong Kong/6013/2014. Results showed that of the three viruses retaining the HA1 158‐160 glycosylation motif (A/Asturias/1951/2014, A/Buenos Aires/6367/2014 and A/Hong Kong/6013/2014), only A/Hong Kong/6013/2014, a virus with NA‐mediated agglutination of RBCs, was able to form plaques in MDCK cells but approximately 100‐ to 1000‐fold less efficiently than in MDCK‐SIAT1 cells. In contrast, A/Israel/O‐7414/2014, a virus with no HA1 158‐160 glycosylation motif which did not display NA‐mediated agglutination of RBCs, formed plaques in both MDCK and MDCK‐SIAT1 cells with equal but low efficiency. A/Hong Kong/7347/2014, a virus with polymorphism at HA1 position 160 resulting in a mixed population of virus with regard to glycosylation at HA1 position 158, showed an intermediate pattern of infection being able to infect MDCK cells approximately 100‐fold less efficiently than MDCK‐SIAT1 cells.

The three 3C.3a viruses also infected MDCK‐SIAT1 cells more efficiently than MDCK cells. However, A/Hong Kong/6315/2014, a virus which like A/Hong Kong/6013/2014 (3C.2a) had acquired polymorphism in the NA gene and was able to agglutinate RBCs through its NA, infected MDCK cells more efficiently than the other two 3C.3a subclade viruses examined, A/Switzerland/9715293/2013 and A/Norway/466/2014, neither of which showed evidence of NA‐mediated agglutination of RBCs.

### 3.4 Analyses of the antigenic properties of viruses in subclades 3C.3a and 3C.2a

As a significant proportion of the subclade 3C.2a viruses were unable to bind to RBCs from a variety of species, MN assays were used for the antigenic characterisation of viruses in the two newly emerged subclades. The majority of tested subclade 3C.2a (53 of 58) and all eight subclade 3C.3a viruses were recognised poorly by a ferret antiserum raised against A/Victoria/361/2011 at titres ≥fourfold lower than for the homologous virus (Table 3). In the light of these results, and others, viruses in both newly emerged subclades differ antigenically from previously circulating A/Victoria/361/2011‐like viruses.

**Table 3:** Summary of antigenic analyses of influenza A(H3N2) viruses—Plaque Reduction Neutralization

Virus used to generate antiserum	Amino acid sequence at HA1 158‐160	Post‐infection ferret antisera								
		A/Victoria/361/2011 (E4/SIAT2)^a^			A/Switzerland/9715293/2013 (SIAT3)^a^			A/Hong Kong/7295/2014 (MDCKx/SIAT3)^a^		
Ferret No.		F09/12			NIB^b^ F13/14			F02/15		
Subclade		3C.1			3C.3a			3C.2a		
		No. of viruses tested	Neutralization titre compared to homologous titre		No. of viruses tested	Neutralization titre compared to homologous titre		No. of viruses tested	Neutralization titre compared to homologous titre	
			≤2‐fold	≥4‐fold		≤2‐fold	≥4‐fold		≤2‐fold	≥4‐fold
3C	NFK	5	5	0	7	5	2	3	0	3
3C.2a	NYT	54	5	49	133	106	27	104	104	0
	NYK	1	0	1	5	3	2	5	5	0
	NYX	1	0	1	10	5	5	11	11	0
	XYT	2	0	2	11	6	5	9	9	0
3C.3	NFK	0	0	0	3	2	1	3	3	0
3C.3a	NSK	8	0	8	36	29	7	29	6	23

a Passage history E=egg, MDCK= conventional MDCK cells, SIAT=MDCK‐SIAT1cells.

b Kindly provided by collaborators at the National Institute for Biological Standards and Controls, UK.

A ferret antiserum raised against a subclade 3C.3a reference virus, A/Switzerland/9715293/2013 propagated in cell culture, recognised 81% of the subclade 3C.3a viruses examined (29 of 36) at titres within twofold of the titre with the homologous virus. This antiserum also recognised most subclade 3C.2a viruses with an intact HA1 158‐160 glycosylation motif (106 of 133, 80%) at neutralisation titres within twofold of that with the homologous virus. Those 3C.2a viruses that had lost or were polymorphic at the glycosylation motif showed somewhat poorer recognition, with 54% of viruses being recognised at titres within twofold of the titre of the antiserum with the homologous virus (14 of 26). Sequencing of the analysed viruses showed that two‐thirds of those recognised at reduced titre (titres ≥fourfold reduced compared to homologous titre) had additional substitutions in HA1, such as Q197R, R261Q, N171K and S114T, that may account for low reactivity irrespective of any loss or polymorphism at the HA1 158‐160 glycosylation motif.

In contrast, a ferret antiserum raised against a cell culture‐propagated subclade 3C.2a virus, A/Hong Kong/7295/2014, recognised all the 3C.2a viruses very well, whether or not they encoded the HA1 158‐160 glycosylation motif, but most subclade 3C.3a viruses tested (23 of 29, 79%) were recognised at titres ≥fourfold lower than the titre with the homologous virus (Table 3).

We next asked specifically whether antisera raised against reference viruses that had retained or lost the HA1 158‐160 glycosylation motif recognised circulating viruses equally well.

Subsets of a panel of subclade 3C.2a viruses were characterised using post‐infection ferret antisera raised against three subclade 3C.2a viruses that differed at the HA1 158‐160 glycosylation motif (A/Hong Kong/7295/2014: NYT, A/Hong Kong/5738/2014: NYT/K, and A/England/530/2014: NYK), in MN assays (Table 4). As shown in Table 3, the antiserum raised against A/Hong Kong/7295/2014, a virus retaining the glycosylation motif, recognised all glycoforms of the 3C.2a viruses tested, while antisera raised against viruses that showed partial loss (A/Hong Kong/5738/2014) or complete loss (A/England/530/2014) of the glycosylation motif recognised, respectively, 98% (45/46) and 85% (23/27) of the test viruses retaining the NYT motif at neutralisation titres within twofold of their homologous titres. Sequence analyses showed that four viruses which had ≥fourfold reductions in neutralisation titres had additional substitutions at other positions in HA1 (D136E, S146G, S230P), possibly accounting for their reduced recognition by the antisera.

**Table 4:** Summary of antigenic analyses of subclade 3C.2a influenza A(H3N2) viruses—Plaque Reduction Neutralization

Virus used to generate antiserum	Subclade	Post‐infection ferret antisera								
Ferret No.		A/Hong Kong/7295/2014 (MDCKx/SIAT3)^a^			A/Hong Kong/5738/2014 (MDCK3)^a^			A/England/530/2014 (MDCK1/SIAT1)^a^		
Amino acid sequence at HA1 158‐160		F02/15			F30/14			F04/15		
		NYT			(N/K)YT			NYK		
		3C.2a			3C.2a			3C.2a		
		No. of viruses tested	Neutralization titre compared to homologous titre		No. of viruses tested	Neutralization titre compared to homologous titre		No. of viruses tested	Neutralization titre compared to homologous titre	
			≤2‐fold	≥4‐fold		≤2‐fold	≥4‐fold		≤2‐fold	≥4‐fold
NYT	3C.2a	104	104	0	46	45	1	27	23	4
NYK		5	5	0	1	1	0	0	0	0
NYX		11	11	0	1	1	0	0	0	0
XYT		9	9	0	2	2	0	1	1	0

a Passage history MDCK=conventional MDCK cells, SIAT=MDCK‐SIAT1 cells.

Taken together, these results indicate that loss or polymorphism of the HA1 158‐160 glycosylation motif in subclade 3C.2a viruses had only a minor effect on the antigenic properties of the viruses whether analysed by antisera raised against the 3C.3a reference virus A/Switzerland/9715293/2013 or against reference subclade 3C.2a viruses with or without the 158‐160 glycosylation motif. Moreover, these results indicate that viruses in both newly emerged subclades are antigenically different from viruses previously in circulation and, while there is some antigenic similarity between viruses in the two subclades, groups of viruses designated by subclades 3C.3a and 3C.2a are not antigenically identical to one another.

### 3.5 Reduced plaque size or abortive infections after further passages at high multiplicity of infection (MOI) in MDCK‐SIAT1 cells

During the course of the MN assays, it was observed that when undiluted culture supernatant from a previous passage was used as inoculum in MDCK‐SIAT1 cells, many viruses produced subpopulations that gave very small plaques despite the presence of significant NA activity in the passage supernatant. Small plaques might be indicative of abortive infection of the cells. To investigate this possibility, a subset of viruses were analysed in detail as they were serially passaged at varying virus dilutions.

**Figure 2:**
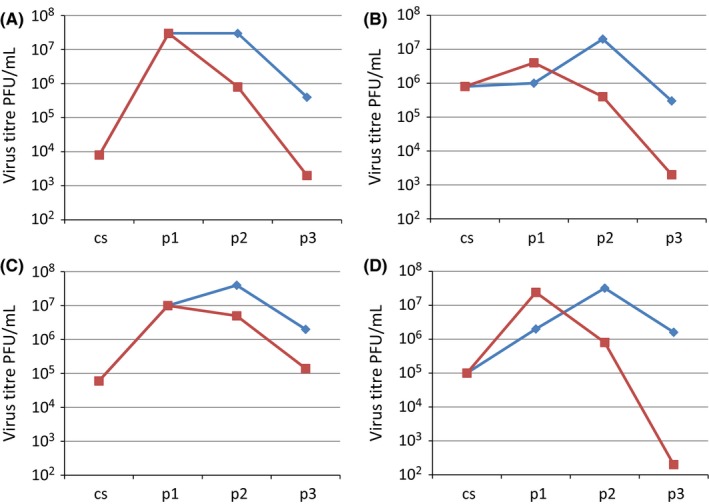
Propagation of recent influenza A (H3N2) viruses in MDCK‐parent and MDCK‐SIAT cells using undiluted culture supernatants serially passed from passage number 1 to 2 and from passage 2 to 3. ( 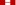 MDCK‐SIAT1 cells, 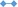 MDCK‐Parent cells). A, A/Hong Kong/6086/2014 and B, A/Hong Kong/6315/2014, both 3C.3a viruses; and C, A/Hong Kong/7127/2014 and D, A/Hong Kong/7364/2014, both 3C.2a viruses. cs=clinical sample, p=passage

Figure 2 shows the infectious titres of virus following serial propagation of subclade 3C.3a (panels A and B) and subclade 3C.2a (panels C and D) viruses in MDCK‐SIAT1 or MDCK cells by transferring 200 μL of cell culture supernatant as inoculum from passage 1 to passage 2, and similarly from passage 2 to 3. Most viruses propagated in MDCK‐SIAT1 cells gave similar or higher virus titres compared to propagation in MDCK cells for the first passage, but the infectious titres attained progressively decreased at passages 2 and 3 during propagation in MDCK‐SIAT1 cells. In contrast, the infectious titres rose at passage two when MDCK cells were used. At passage three, the infectious titres of viruses propagated in MDCK‐SIAT1 cells were considerably lower than those from MDCK cells.

**Figure 3:**
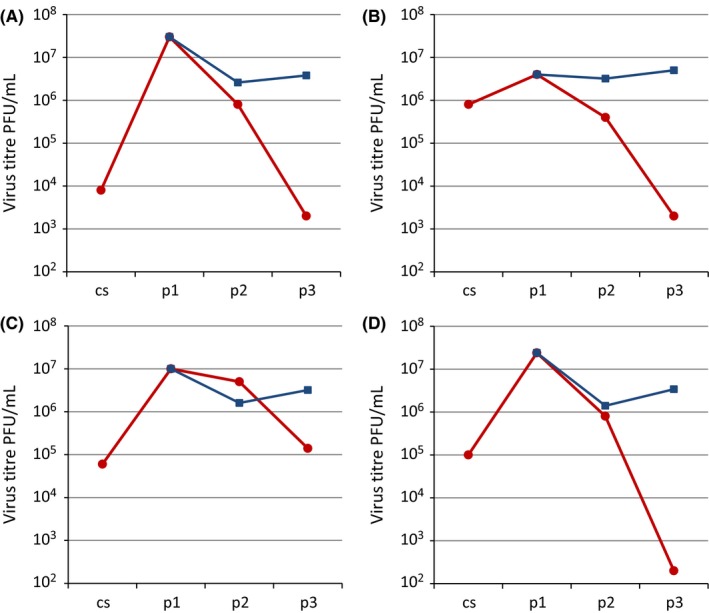
Comparison of virus yield with different dilutions of inoculum for recent H3N2 viruses in MDCK‐SIAT1 cells. A, A/Hong Kong/6086/2014 and B, A/Hong Kong/6315/2014, both 3C.3a viruses; C, A/Hong Kong/7127/2014 and D, A/Hong Kong/7364/2014, both 3C.2a viruses. 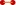 Virus passed using undiluted culture supernatants, 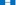 Virus passed at 10^−6^ dilution of the inoculum from passage 1 to passage 2, and at 10^−5^ dilution from passage 2 to passage 3. cs=clinical sample, p=passage

In the light of the falling titres on passage of viruses in MDCK‐SIAT1 cells, a comparison of the yield of virus propagated at higher dilutions of inocula in MDCK‐SIAT1 cells is shown in Figure 3. The yield of infectious virus was assessed for samples diluted by six logs at passage 2 and by five logs at passage 3 prior to transfer onto the next culture flask. The results show that passage at the higher dilutions retained the titres of infectious virus. The NA activity of the recovered viruses was also measured as an indicator of the number of virus particles present in the culture. In contrast to the loss in virus infectivity, notably so by the third passage (Figure S1), the NA activity was less markedly reduced when virus was passed at the higher multiplicity. The NA activity to virus infectivity ratio served as a proxy for the particle to infectivity ratio, and the ratio increases observed implied that some form of defective particles were being generated on passage at high multiplicity in MDCK‐SIAT1 cells.

Next, to examine whether serial propagation of virus at high dilution affected the virus sequence polymorphism that might emerge, with particular reference to the HA1 158‐160 glycosylation motif acquired by the 3C.2a viruses, samples of culture supernatant at each passage at the two dilutions were examined by Sanger sequence analysis (Table S2). Very little polymorphism was observed for viruses passaged at high MOI, but for viruses passaged at a much lower MOI, a higher degree of polymorphism was detected in the HA. This was notably so for viruses in the 3C.2a subclade, where all five viruses showed some polymorphism in the HA and three of the five viruses showed polymorphism at residue 159 in the HA1 158‐160 glycosylation motif. The significance of this is uncertain. An intermediate MOI of 0.4×10^−2^ to 6×10^−2^ retained faithful virus replication (Table S2).

## 4 Discussion

The common features of viruses in two newly emerged H3N2 HA subclades, 3C.2a and 3C.3a, are changes in the glycosylation and receptor‐binding sites of HA. In comparison with the previously circulating A/Victoria/361/2011‐like and A/Texas/50/2012‐like viruses, HAs in both subclades have an HA1 N225D substitution, located in the 220 loop of the receptor‐binding pocket, and changes at HA1 position 159 located in antigenic site B adjacent to the receptor‐binding pocket. Our previous study^1^ showed that D225N substitution in viruses isolated after 2004 was responsible for the loss of binding to turkey RBCs. Here, we show that the N225D reversion in subclade 3C.3a and 3C.2a viruses was unable to restore this binding. Moreover, viruses in both subclades have altered glycosylation potential compared to previously circulating H3N2 viruses: subclade 3C.2a viruses have lost and gained potential glycosylation motifs due, respectively, to N144S and K160T substitutions in HA1, and viruses in subclade 3C.3a have lost a potential glycosylation motif as a result of T128A substitution in HA1. Taken together, these changes potentially affect not only the antigenic but also the receptor‐binding properties of the new viruses and impact on faithful virus propagation in culture.

We have shown here that propagation of the new H3N2 viruses in conventional MDCK cells continues to promote NA‐mediated agglutination of RBCs in a significant number of viruses from subclades 3C.2a and 3C.3a. Consistent with NA‐mediated agglutination of RBCs, sequence analysis of four of five viruses in subclade 3C.3a showed polymorphism at residue 151 of the NA while eight of nine 3C.2a viruses examined in detail showed either substitution or polymorphism at residues 148 or 151 of the NA, these changes being associated with NA‐mediated agglutination of RBCs^6^. In contrast, no such selection was seen when viruses in the new subclades were propagated in MDCK‐SIAT1 cells. We also observed that viruses from both subclades generally more readily infected MDCK‐SIAT1 cells compared to conventional MDCK cells, whether complemented by the NA or not. From these results, we can conclude that the recent H3N2 viruses in subclades 3C.2a and 3C.3a, with HA genes once again encoding HA1 225D, have not overcome the previously identified difficulties of viruses collected since 2005 to propagate faithfully in conventional MDCK cells; thus, the interpretation of antigenic data for viruses in the new subclades is likely to remain problematic^6^.

We observed loss of the glycosylation motif at HA1 158‐160 in subclade 3C.2a viruses in four of the eight HA sequences at passage 3 when viruses were propagated in conventional MDCK cells but, importantly, similar changes were not observed in viruses propagated in MDCK‐SIAT1 cells. Glycosylation was stable in subclade 3C.3a viruses in both cell lines.

To examine the antigenic relationships between viruses in the two new subclades, and the influence of HA and NA amino acid substitutions and polymorphism acquired during culture, we used MN assays^14^. This choice was necessitated because many 3C.2a viruses did not agglutinate RBCs from any species examined here. Using post‐infection ferret antisera, we concluded that viruses in both newly emerged subclades differ antigenically from previously circulating H3N2 viruses and, while there is some antigenic similarity between viruses in the two subclades, groups of viruses in each subclade can be distinguished antigenically. These conclusions are supported by our unpublished data considered during the development of WHO recommendations on influenza vaccines for use in the 2016 Southern Hemisphere and 2016‐2017 Northern Hemisphere influenza seasons^15^, ^16^. These conclusions are also consistent with those adduced by Chambers et al.^17^ using a ferret antiserum and sheep hyperimmune antisera.

In the light of the observation of the loss of the HA1 158‐160 glycosylation motif during culture in conventional MDCK cells, we asked whether this might be reflected in antigenic analysis of circulating viruses. For this, we used three antisera raised against viruses in genetic group 3C.2a that had either retained, or had lost, or was polymorphic for this glycosylation motif, and analysed examples of viruses from subclade 3C.2a, propagated in cell culture, that had similar motif compositions. From MN assay results, we concluded that antisera raised against the three reference viruses generally recognised test viruses with the three motif compositions similarly and that the antigenic properties of viruses that had acquired polymorphism at HA1 158‐160 are similar to those with an intact glycosylation motif at this site. It is noteworthy that the virus recommended as the prototype for use in influenza vaccines for the 2016 Southern Hemisphere and 2016‐2017 Northern Hemisphere seasons, A/Hong Kong/4801/2014, had lost its HA1 158‐160 glycosylation motif during adaptation to propagation in hens’ eggs.

The observations made here support recommending continued use of the MDCK‐SIAT1 cell line for propagation of current H3N2 viruses, although this is not free of problems. In MN assays, in some cases, we observed virus plaques of very small size and as virus was passaged in the MDCK‐SIAT1 cell line, infectivity was progressively lost although infectivity could be maintained by passage at lower MOI (Figure 3). This is probably due to accumulation of defective viruses as NA activity remained high when infectivity was lost on virus passage at an MOI in the order of 1 to 10 MDCK‐SIAT1 cell PFU/cell (an MOI often used to retain HA activity on passage, akin to the retention of virus sialidase activity) (Figure S1). However, viruses propagated at low MOI in MDCK‐SIAT1 cells, while retaining infectivity, carried HA and NA gene sequences showing increased polymorphism compared with viruses propagated at higher MOIs. The effect of the appearance of apparently defective virus was greater in MDCK‐SIAT1 cells than in conventional MDCK cells. This is likely to be linked to the observed higher infection rate of MDCK‐SIAT1 cells compared with MDCK cells (Figure 1) by the current H3N2 viruses, effectively increasing the MOI, and thereby fostering the generation of defective viruses in culture.

To sum up, from the perspective of avoiding the selection of polymorphic or mutant viruses, whether mutated in the HA gene and/or the NA gene, we conclude that the current H3N2 viruses in subclades 3C.2a and 3C.3a remain more faithfully propagated in the MDCK‐SIAT1 cell line than in conventional MDCK cells. This is despite amino acid substitutions in the vicinity of the receptor‐binding site, notably at residue 225, which might have overcome the poor efficiency of H3N2 viruses circulating since 2005 to infect conventional MDCK cells^1^. However, passage of the current H3N2 subclade 3C.2a and 3C.3a viruses in the MDCK‐SIAT1 cell line can lead to the loss of infectivity associated with a postulated increase in the accumulation of defective virus particles—the postulate being based on the retention of NA activity despite low infectivity as a proxy for the particle to infectivity ratio. To overcome this problem, we submit that current H3N2 viruses should be cultured in the MDCK‐SIAT1 cell line to maintain faithful replication of the virus, and at an appropriate MOI to retain infectivity and avoid the generation of defective viruses. This is critical if HI and MN assays are to remain the primary, universally employed methods for antigenic characterisation of H3N2 viruses.
