# Supplementary material for: The characteristics and antigenic properties of recently emerged subclade 3C.3a and 3C.2a human influenza A(H3N2) viruses passaged in MDCK cells
Source: Influenza Other Respir Viruses. 2017 Feb 28;11(3):263–74. doi: 10.1111/irv.12447 (PMC5410720; doi:10.1111/irv.12447)
Supplement: Supplementary file 1 [file IRV-11-263-s001.docx]

10^8^

10^7^

10^6^

10^5^

10^4^

10^3^

10^2^

10^7^

10^6^

10^5^

10^4^

10^3^

10^2^

**B**

**A**

10^7^

10^6^

10^5^

10^4^

10^3^

10^2^

10^8^

10^7^

10^6^

10^5^

10^4^

10^3^

10^2^

**D**

**C**

Fig. S1 Correlation between the neuraminidase activity and infectivity propagated in MDCK-SIAT1 cells

Titre of virus (PFU/ml) are shown in bars and are from figure 3, NA activity estimated by MUNANA IC 50 dilution factor are shown in red dot. Virus passed using undiluted culture supernatants as described in Figure 2, Virus passed at 10^-6^ dilution of the inoculum from passage 1 to passage 2, and at 10^-5^ dilution from passage 2 to passage 3, cs = clinical sample
